# Structural and Antigenic Variation among Diverse Clade 2 H5N1 Viruses

**DOI:** 10.1371/journal.pone.0075209

**Published:** 2013-09-27

**Authors:** David A. Shore, Hua Yang, Amanda L. Balish, Samuel S. Shepard, Paul J. Carney, Jessie C. Chang, Charles T. Davis, Ruben O. Donis, Julie M. Villanueva, Alexander I. Klimov, James Stevens

**Affiliations:** Influenza Division, Centers for Disease Control and Prevention, Atlanta, Georgia, United States of America; Center for Biologics Evaluation and Research, United States of America

## Abstract

Antigenic variation among circulating H5N1 highly pathogenic avian influenza A viruses mandates the continuous production of strain-specific pre-pandemic vaccine candidates and represents a significant challenge for pandemic preparedness. Here we assessed the structural, antigenic and receptor-binding properties of three H5N1 HPAI virus hemagglutinins, which were recently selected by the WHO as vaccine candidates [A/Egypt/N03072/2010 (Egypt10, clade 2.2.1), A/Hubei/1/2010 (Hubei10, clade 2.3.2.1) and A/Anhui/1/2005 (Anhui05, clade 2.3.4)]. These analyses revealed that antigenic diversity among these three isolates was restricted to changes in the size and charge of amino acid side chains at a handful of positions, spatially equivalent to the antigenic sites identified in H1 subtype viruses circulating among humans. All three of the H5N1 viruses analyzed in this study were responsible for fatal human infections, with the most recently-isolated strains, Hubei10 and Egypt10, containing multiple residues in the receptor-binding site of the HA, which were suspected to enhance mammalian transmission. However, glycan-binding analyses demonstrated a lack of binding to human α2-6-linked sialic acid receptor analogs for all three HAs, reinforcing the notion that receptor-binding specificity contributes only partially to transmissibility and pathogenesis of HPAI viruses and suggesting that changes in host specificity must be interpreted in the context of the host and environmental factors, as well as the virus as a whole. Together, our data reveal structural linkages with phylogenetic and antigenic analyses of recently emerged H5N1 virus clades and should assist in interpreting the significance of future changes in antigenic and receptor-binding properties.

## Introduction

Highly pathogenic avian influenza (HPAI) A (H5N1) viruses have caused severe respiratory and systemic disease in humans and feature an exceptionally high mortality rate. Continual outbreaks of HPAI H5N1 viruses among poultry [1]–[3] represent a constant threat for direct inter-species transmission to humans. More than 600 cases of human infection with H5N1 viruses have been confirmed over the past decade [4] and, although widespread transmission of these viruses among the human population has yet to be reported, isolated cases of probable human-to-human transmission have been identified [5]–[8]. With continuous outbreaks of H5N1 virus among wild and domestic bird populations across Asia, Europe, the Middle East and Africa, it is clear why an H5N1 virus capable of sustained human transmission remains a global public health concern [9].

All influenza viruses undergo frequent mutation in their surface proteins, hemagglutinin (HA) and neuraminidase (NA), resulting in antigenic variation among circulating strains [10]. H5N1 viruses have evolved rapidly whilst circulating among bird populations since 1996, and changes to the H5 HA gene have been used as a basis for phylogenetic characterization of a growing number of distinct viral subgroups referred to as clades 0–9 [11]. The constant evolution of viruses within each subgroup has generated multiple second, third and fourth order clades defined by their phylogenetic clustering and genetic distance [11], [12]. Currently, the majority of viruses circulating worldwide belong to clades 1 and 2, with clades 1.1, 2.1.3.2, 2.2.1, 2.2.2, 2.3.2.1, 2.3.4 and 2.3.4.2 responsible for recent human infections [13]–[16].

H5N1 viruses possess a hemagglutinin against which the human population is immunologically naïve [17], [18], and thus, the World Health Organization (WHO) prepares for the possibility of a future H5N1 pandemic by coordinating the development of candidate vaccine seed viruses from circulating strains [15]. The rapid evolution of H5N1 viruses gives rise to changes in the HA protein that result in altered antigenicity and prevent effective long-term immunity from strain-specific vaccination [19]. To account for this variation in antigenicity, the WHO has 22 H5N1 vaccine seed viruses currently available and 2 more in production or pending safety evaluation [20].

In addition to changes in antigenicity, changes in the receptor-binding characteristics of circulating H5 viruses have also become the focus of surveillance and pandemic preparedness efforts [21]. Influenza A viruses infect both avian and mammalian species through binding of HA to sialic acid (Neu5Ac) moieties on the surface of host cells, and viral host specificity is determined by both viral HA structure and the type of host receptor [22]. Avian influenza viruses preferentially bind α2-3-linked Neu5Ac receptors present in the intestinal epithelia of birds, while human-adapted viruses preferentially bind α2-6-linked Neu5Ac within the upper airway [23]–[25]. Changes among H5N1 viruses which enhance binding to the human-type Neu5Ac-α2-6-Gal linkage are considered a major factor in the generation of viruses with the capacity for human to human transmission [26]–[28] and, as such, represent potentially critical features of emerging strains with pandemic potential.

Crystallographic analyses of hemagglutinins from distinct clades of H5N1 viruses can provide important insight into changes in the antigenic and receptor-binding properties of emerging strains. However, structural information is currently limited to only clade 1 viruses, which emerged more than 8 years ago and whose progeny are known to circulate only in the Mekong Delta regions of Vietnam and Cambodia [29], [30]. Herein, we report the antigenic properties, receptor-binding specificities, and crystal structures of hemagglutinins of three different H5N1 clades responsible for recent fatal human infections in geographically distinct areas: A/Egypt/N03072/2010 (Egypt10, clade 2.2.1), A/Hubei/1/2010 (Hubei10, clade 2.3.2.1) and A/Anhui/1/2005 (Anhui05, clade 2.3.4) [31], [32]. These viruses are phylogenetically diverse and have distinct antigenic profiles, leading to their selection by the WHO as pre-pandemic vaccine candidates [16].

## Materials and Methods

### Cloning, Expression and Purification of Recombinant HA Proteins

All three proteins were derived from reverse genetics constructs cloned from the parental viruses (A/Hubei/1/2010 (Hubei10, clade 2.3.2.1); A/Egypt/N03072/2010 (Egypt10, clade 2.2.1); A/Anhui/1/2005 (Anhui05, clade 2.3.4). The HA gene of each clone was modified at the HA1/HA2 cleavage site to remove the polybasic sequence (Table S2) [64]. A/Hubei/1/2010 (Hubei10, clade 2.3.2.1) was received from CDC China as a genetically modified clone, also lacking the polybasic cleavage motif. All three constructs were cloned into the baculovirus transfer vector, pAcGP67-A (BD Biosciences, San Jose, CA), and utilized a thrombin site at the C-terminus of Anhui05 followed by a trimerizing sequence (foldon) from the bacteriophage T4 fibritin for generating functional trimers [65], and a His-Tag to aid purification [66]. Transfection and virus amplification were carried out as described previously [67], [68].

Egypt10 and Hubei10 proteins were expressed from High Five™ cells (Life Technologies, Grand Island, NY) in 10-stack CellSTACK™ culture chambers (Corning Inc., Corning, NY). Soluble HA proteins were recovered from the culture supernatant by His-Tag purification and subjected to thrombin cleavage and gel filtration chromatography. Purified trimeric proteins were buffer exchanged into 10 mM Tris-HCl, 150 mM NaCl, pH 8.0 and concentrated to 12 mg/ml for crystallization trials. At this stage, the protein sample still contained the additional plasmid-encoded residues at both the N (ADPG) and C terminus (SGRLVPR). Purified Anhui05 protein (5 mg; Catalog No. FR-86) was obtained from the Influenza Reagent Resource (http://www.influenzareagentresource.org). This was subjected to thrombin digestion and purification as described for the other two proteins and was concentrated to 10 mg/ml for crystallization trials.

### Crystallization and Data Collection

For all three proteins, initial crystallization trials were set up using a Topaz™ Free Interface Diffusion (FID) Crystallizer system (Fluidigm Corporation, San Francisco, CA). Anhui05 crystals were observed in several conditions containing various molecular weight PEG polymers. Following optimization, diffraction quality crystals were obtained at 20°C using a modified method for micro-batch under oil [69], by mixing the protein with a reservoir solution containing 20% PEG 4000 and 100 mM Tris-HCl pH 7.8. Useable crystals of Hubei10 were produced using the sitting drop vapor diffusion method at 20°C within a well containing 0.5 µl protein solution at 12 mg/ml and an equal volume of reservoir solution containing 20% PEG 2000 MME and 100 mM Tris-HCl pH 7.0. Useable crystals Egypt10 were produced using the sitting drop vapor diffusion method at 20°C in a well containing 0.5 µl protein solution at 12 mg/ml and an equal volume of reservoir solution containing 25% PEG 3350 and 100 mM Tris-HCl pH 8.5. Crystals were flash-cooled at 100 K. Datasets were collected at the Argonne National Laboratory Advanced Photon Source (APS) beamlines 22-ID at 100 K. Data were processed with the DENZO-SACLEPACK suite [70]. Statistics for data collection and refinement are presented in Table S1.

### Structure Determination and Refinement

All model building and refinement was carried out using Coot [71], Phenix [72] as well as REFMAC5 [73] using TLS refinement [74]. Model validation was carried out using MolProbity [75]. H5 HA from Anhui05 (clade 2.3.4) HA crystallized in trigonal space group *P*3 and the crystal structure was determined by molecular replacement with Phaser [76], using PDB:2FK0 as search model, to 2.7 Å resolution. Three monomers that each form one-third of an independent crystallographic trimer occupy the asymmetric unit with an estimated solvent content of 59% based on a Matthews’ coefficient (*Vm*) of 3.01 Å^3^/Da. The three HA monomers within the ASU are highly similar and all Ca atoms superimpose with an RMSD of only 0.7 Å.

H5 HA from Egypt10 (clade 2.2.1) crystallized in trigonal space group *H*3 and the crystal structure was determined by molecular replacement with Phaser [76], using PDB:2FK0 as search model, to 2.5 Å resolution. The crystallographic asymmetric unit (ASU) contains 4 monomers (A/B, C/D, E/F and G/H), which are all highly similar to each other (Ca atoms superimpose with an RMSD of only 0.3 Å), with all significant differences between monomers restricted to flexible loops, due to differential packing of the molecules within the ASU. Of note, residues 125–141 form a highly exposed loop that is disordered in 3 of the 4 molecules within the ASU. This loop, which forms a component of the conserved receptor-binding domain, is only visible within continuous 2F_O_-F_C_ electron density of monomer A/B.

HA from Hubei10 (clade 2.3.2.1) HA crystallized in monoclinic space group *C2* and the crystal structure was determined by molecular replacement with Phaser [76], using PDB:2FK0 as search model, to 2.6 Å resolution. The crystallographic ASU contains one trimer. Each monomer in the trimer (A/B, C/D and E/F) is highly similar to the others, with their Cα atoms superimposing with an RMSD of only 0.4 Å.

### Glycan Binding Analyses

Glycan microarray printing and recombinant HA analyses have been described previously [30], [48], [53], [68], [77]. Imprinted slides were produced under contract using the Consortium for Functional Glycomics glycan library (CDC version 1 slides; see Table S1 for glycans used in these experiments). For kinetic studies, biotinylated glycans, Neu5Ac(α2–3)Gal(β1–4)Glc-biotin (3SLN-b), Neu5Ac(α2–3)Gal(β1–4)GlcNAc(β1,3)Gal(β1–4)GlcNAcb-biotin (3SLNLN-b) and Neu5Ac(α2–6)Gal(β1–4)GlcNAc(β1,3)Gal(β1–4)GlcNAcb-biotin (6SLNLN-b), obtained from the Consortium for Functional Glycomics (www.functionalglycomics.org) through the resource request program, were coupled to streptavidin coated biosensors (Fortebio Inc.). Recombinant HA was diluted to 1 mg/ml (4.42 µM) trimer in kinetics buffer (PBS containing 0.02% (vol/vol) Tween 20, 0.005% (vol/vol) sodium azide and 100 µg/ml bovine serum albumin). Binding was analyzed by Bio-Layer Interferometry (BLI) on an Octet Red instrument (Fortebio, Inc.) according to the manufacturers instructions and data were analyzed using the system software and fitted to a 1∶1 binding model.

### Hemagglutination Inhibition Assay

Influenza A (H5N1) viruses were antigenically characterized in the hemagglutination inhibition (HI) assay using post-infection ferret antiserum. Wild-type viruses of A/Egypt/N03072/2010 (Egypt/10, clade 2.2.1), A/Anhui/1/2005 (Anhui/05, clade 2.3.4) and A/Vietnam/1203/2004 (Viet/04) were inoculated into 10 day-old embryonated chicken eggs and allantoic fluid containing virus was harvested from eggs within 48 hours [78]. CDC Institutional Animal Care and Use Committee (IACUC) approval was not required for virus propagation in embryonated chicken eggs because all eggs were destroyed prior to hatching. All work was carried out according to guidance from the Office of Laboratory Animal Welfare (OLAW), National Institutes of Health, who is responsible for implementation of the PHS Policy Animal Welfare Act (7 U.S.C. Sections 2131–2159) and the Public Health Service Policy on Humane Care and Use of Laboratory Animals (http://grants.nih.gov/grants/olaw/faqs.htm#App_4).

The A/Hubei/1/2010 (Hubei10, clade 2.3.2) virus was a reassortant, comprising the internal genes A/Puerto Rico/8/1934 (H1N1; PR8) in combination with HA and NA genes derived by reverse genetics (RG) methods [64]. Clones for the HA and NA genes were provided by CDC China as a genetically modified clone, lacking the polybasic cleavage motif. Ferret antiserum was generated by intranasal inoculation, boosting at day 14, with ≥500 HA units of concentrated virus and Titermax as adjuvant. To obtain concentrated virus, 5 ml of infected allantoic fluid was subjected to ultracentrifugation (45,000×*g* at 4°C for 3 hours). The virus pellet was re-suspended in 300 µl PBS and tested to confirm titers of ≥500 HA units. After 14 days post-boost sera was collected. Individual ferret sera corresponding to each virus tested were generated in at least two animals (i.e. at least two separate lots were produced for each virus). The homologous titer of each sera was tested and confirmed to be within 2-fold of other lots produced against the same virus. If a greater than 2-fold difference in homologous titers was determined, these sera were not used in the HI analysis. All ferret work was conducted in an Association for Assessment and Accreditation of Laboratory Animal Care International-accredited animal facility, under the guidance of the Centers for Disease Control and Prevention’s Institutional Animal Care and Use Committee. HI assays were performed using turkey erythrocytes as previously described [78], [79]. All work was performed in a Biosafety Safety level 3 enhanced facility.

### H5N1 Clade Sequence Analysis

Nucleotide sequences for the H5 hemagglutinins were obtained from GISAID (www.gisaid.org), Genbank (www.ncbi.nlm.nih.gov/genomes/FLU), and the WHO website (www.who.int/influenza/gisrs_laboratory/h5n1_nomenclature) using a subset (no outliers) of the annotated H5 dataset described in [80]. Sequence accession number, strain names, clade annotations, and data sources are given in Supporting File 1 along with a GISAID acknowledgement table. Sequences were aligned and trimmed (JalView, [81]to the mature HA1 region (including cleavage site) and samples less than 90% of the alignment length were removed via a custom Perl script (available upon request). Alignments were made using MAFFT [82] and MUSCLE [83] and a translated consensus sequence for each clade was calculated using Geneious Pro version 5.5.6 (http://www.geneious.com).

### PDB Accession Codes

The atomic coordinates and structure factors of Anhui05, Egypt10 and Hubei10 HA are available from the RCSB PDB under accession codes 4 KWM, 4 KW1 and 4 KTH.

## Results

### Antigenic Properties of HPAI H5N1 Viruses from Distinct Clades

Candidate influenza vaccine viruses are primarily selected based upon differences in the antigenic properties of circulating strains, as determined by the hemagglutination inhibition (HI) assay. To assess the antigenic profiles of the Egypt10, Hubei10, and Anhui05 vaccine candidates, we compared the HI cross-reactivity of these viruses to one another and to the well-characterized clade 1 candidate vaccine virus, A/Vietnam/1203/2004 (Viet04), using strain-specific polyclonal ferret antisera. Comparison of the serum HI titers of these phylogenetically diverse viruses [33] indicated the extent to which they were antigenically related (see Table 1 and Table S1). Based on the two-way cross-reactive HI titers, a majority of the viruses tested were ≥8-fold down when compared to sera generated against heterologous viruses. An exception was Egypt10 (clade 2.2.1), which was covered <8-fold, compared to homologous titers, by antisera against the three other viruses. Conversely, however, the Egypt10 antisera produced a >8-fold reduction in homologous versus heterologous titers relative to the three other viruses (Table 1 and Table S1). Viet04 antigen had reduced cross-reactivity with antisera from heterologous viruses, but its antisera did consistently cross-react with Anhui05 and Egypt10. Anhui05 and Hubei10 displayed more straight-forward two-way patterns wherein 8-fold or greater reduction in cross-reactivity was measured.

**Table 1:** Antigenic and amino acid sequence variation among different clades of H5N1 vaccine candidate viruses.

		Viet04 (1)	Anhui05 (2.3.4)	Egypt10 (2.2.1)	Hubei10 (2.3.2.1)
**HA Antigen**	**clade**	**HI Titers (antisera)** a			
Viet04	1	285 b	≥8^c^	≥8	<8
Anhui05	2.3.4	<8	718	≥8	≥8
Egypt10	2.2.1	<8	<8	3620	<8
Hubei10^d^	2.3.2.1	≥8	≥8	≥8	285
**HA**		**HA1% sequence identity (%)** e			
Viet04	1	100			
Anhui05	2.3.4	94	100		
Egypt10	2.2.1	93	93	100	
Hubei10	2.3.2.1	91	92	91	100
**HA**		**% sequence identity of surface residues only (total # differences)** f			
Viet04	1	100			
Anhui05	2.3.4	95 (9)	100		
Egypt10	2.2.1	91 (15)	93 (12)	100	
Hubei10	2.3.2.1	89 (19)	90 (16)	89 (18)	100

a Hemagglutination inhibition (HI) titers were determined using turkey red blood cells.

b Titers for homologous antigen/antisera are shown with values underlined. Titers are presented as the geometric mean titers (GMT) calculated from five independent HI tests.

c Differences among strain-specific cross reactivity are quoted as dilutions relative to that of the end-point dilution value for the homologous antigen/sera response. Viruses are considered antigenically diverse if titers are reported as ≥8-fold difference in two-way tests.

d Only a reassortant virus for Hubei10 was used in assay. Others were wild type viruses.

e Amino acid sequence identities were calculated for the 267 residues of the HA1 structural domain (residues 34–300) of the mature HA, using CLUSTALX [84].

f Surface residue differences were quantified for the HA1 structural domain (residues 34–300) of the mature HA monomer. For this analysis, 166 of the 267 residues were considered surface residues. The number of surface residue substitutions is given in parentheses.

HI reactivity is primarily dependent upon the inhibition of receptor-binding by antibodies that recognize the globular head region of the HA1 subunit, and variation in the HI cross-reactivity titers of viruses is attributed primarily to differences in surface-exposed residues of the HA1 [34]. A comparison of the amino acid sequence identity indicated that these H5N1 HAs share a high degree of sequence homology (91–94%). Furthermore, the number of changes among surface-exposed residues in the HA1 of each virus is consistently low, ranging from 9 between Anhui05 and Viet04, to 19 between Hubei10 and Viet04 (Table 1). These findings highlight the fact that sequence identity across the surface of the HA protein *per se* is an insufficient measure for determining antigenic relatedness among these H5 clades and imply that antigenicity is associated with only a small subset of residues.

### Crystal Structures of Clades 2.2.1, 2.3.2.1 and 2.3.4 HA Proteins

To determine the structural consequences of evolutionary changes among these diverse H5N1 viruses, we crystallized recombinant HA (recHA) proteins from Egypt10, Hubei10, and Anhui05. All three H5 recHA proteins were amplified from template genes modified for vaccine production, whereby their polybasic cleavage site at positions 320–325 had been removed (Figure S1) [35]. The recHAs all crystallized as trimers and diffraction datasets were collected from native protein crystals of each HA, as described in the methods. All three H5 HAs exhibited a high degree of amino acid sequence identity to Viet04 (∼95% across the entire HA), and the three crystal structures were determined by the molecular replacement method, using the trimeric structure of Viet04 HA (PDB:2FK0) as a search model [36]. Data collection and refinement statistics are summarized in Table S2. Amino acid residues in each structure are numbered consecutively, according to the ectodomain fragments of the mature HA1 and HA2 subunits, respectively.

The overall structures of the three H5 HAs are similar to that of Viet04, comprising a trimer of HA0 protomers, each with a globular head containing the receptor-binding site (RBS), a vestigial esterase domain, and a membrane-proximal domain with its distinctive, central helical stalk and HA1/HA2 cleavage site. Although the three HAs were crystallized as the intact HA0 pre-cleaved form, a lack of interpretable electron density for the cleavage site in all three structures implied this region forms an exposed and highly flexible loop that does not influence the structure of the adjacent HA1 and HA2 domains. All three HAs possess *N-*carbohydrates attached to Asn residues at glycosylation sites within the HA1 subdomain. *N-*carbohydrates were clearly visible within the electron density at three of four sites in Egypt10 (Asn11, Asn23 and Asn164; mature H5 HA numbering), at all four glycosylation sites within Hubei10 (Asn11, Asn23, Asn165 and Asn286) and at a single site in Anhui05 (Asn165). Furthermore, *N-*carbohydrates were observed attached to the conserved Asn154 in the HA2 of both Hubei10 and Anhui05. Occupancy at any given *N-*glycosylation site varied among the different molecules within the asymmetric unit in all three structures.

**Figure 1:**
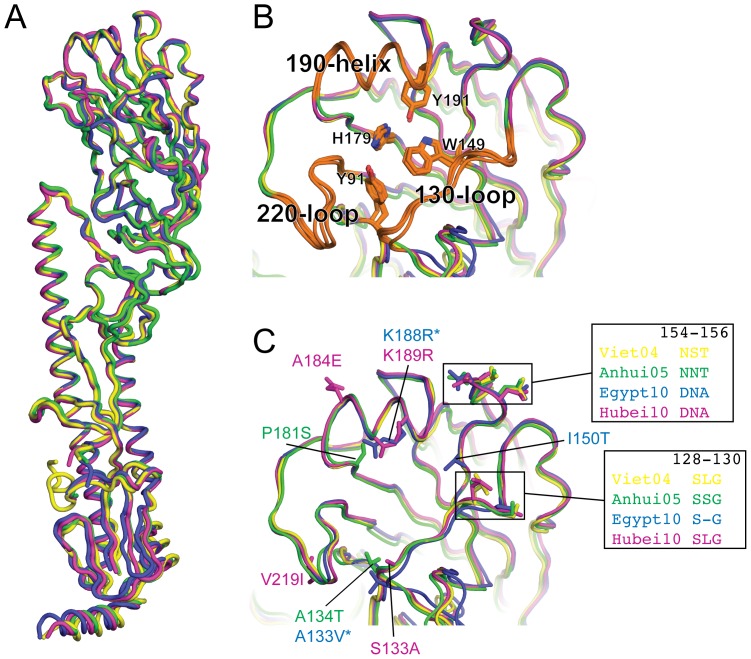
Structural comparison between H5 hemagglutinins. (A) Structural alignment of Anhui05 (green), Egypt10 (blue) and Hubei10 (purple) onto Viet04 (yellow) reveals how structurally related these clades are. (B) Alignment of the receptor-binding site (RBS) reveals conserved structural features and residues. (C) Compared to Viet04, a total of eleven residue differences in and around the RBS are present. Amino acid residues in each structure are numbered consecutively according to the ectodomain fragment of the mature HA1 protein. *Deletion of Leu129 in Egypt10 produces a shift in the numbering of residues 129–324 in Egypt10 relative to structurally equivalent residues in Anhui05 and Hubei10.

To assess structural variations among these HAs, we compared the HA1/HA2 protomer of each structure with that of the Viet04 virus. All Cα atoms in the HA1/HA2 protomers of Egypt10, Hubei10 and Anhui05 superimposed with Viet04 to give root mean square deviations (RMSD) of only 0.86 Å, 0.74 Å and 1.04 Å, respectively, indicating a high degree of structural homology in the configuration of these HAs (Figure 1A & Table S3). All three structures revealed a similar canonical configuration for the membrane-distal receptor-binding site (RBS). Highly conserved residues (Tyr91, Trp149, His179, and Tyr191) formed the base of the sialic acid binding pocket (Figure 1B), surrounded by three highly-conserved structural elements: a 190-helix, a 220-loop, and a 130-loop. A profound difference in the amino acid sequence of the RBS in Egypt10 is a deletion of Leu129 coupled with the substitution of Ile151Thr (del129/Thr151), a characteristic found among the majority of circulating clade 2.2.1 viruses isolated in Egypt [3] and reminiscent of the HA from the 1957 H2N2 pandemic strain [37]. The structural consequence of this deletion/substitution combination is restricted to a minimal rearrangement of residues 128 and 130. There was no perceived effect on the overall conformation of the 130-loop and no change in the association of Ser128 or Gly130 with Thr151 relative to the other H5 structures (Figure 1C).

### Effect of Structural Changes on Antigenicity

**Figure 2:**
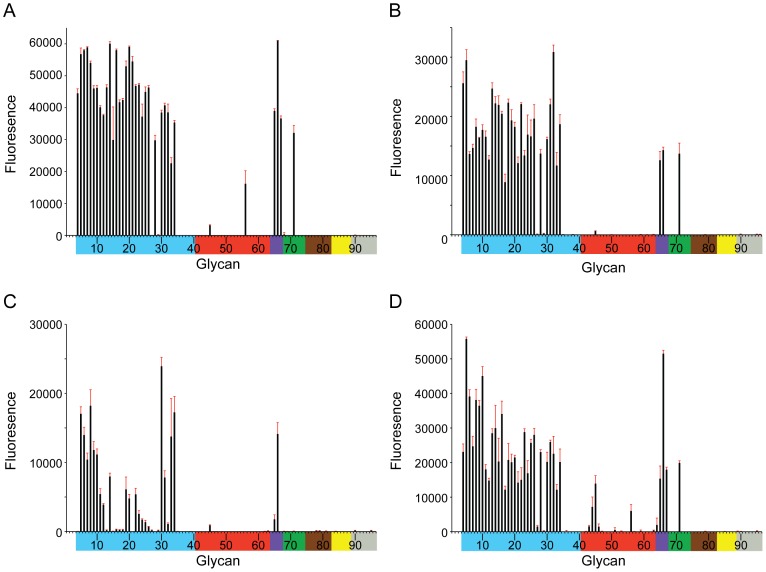
Structural variation among the different H5N1 clades. (A) Surface representation of the Hubei10 trimeric HA indicating the positions of surface exposed residue substitutions among Clade 1 (Viet04), clade 2.3.4 (Anhui05), clade 2.2.1 (Egypt10) and clade 2.3.2.1 (Hubei10). Positions containing single substitutions are colored cyan and positions containing multiple substitutions are colored magenta. (B) Amino acid consensus sequences of H5N1 HA clades at positions equivalent to the HA antigenic sites, Ca, Cb, Sa and Sb, of human H1N1 viruses [39], are shown. Clade 1 (Viet04), clade 2.3.4 (Anhui05), clade 2.2.1 (Egypt10) and clade 2.3.2.1 (Hubei10) are highlighted in red. Structural positions of these equivalent sites are highlighted on the Hubei10 trimeric structure (Ca; pale yellow, Cb; wheat, Sa; pale green, Sb; pale blue). Asparagine residues that are potentially N-glycosylated are colored orange.

The structures of antigenically diverse H5 HAs highlight solvent-accessible residues unique to each strain that can give rise to diversity in the humoral immune response. Compared to the clade 1 Viet04 structure, residue substitutions among these three viruses are restricted to 41 positions, of which only 29 are surface exposed (see Table S4). The spatial distribution of these residues is highlighted in Figure 2. Residue substitutions in the vicinity of the membrane-distal RBS of HA may influence HI reactivity and thereby impact the antigenic profile of the virus [38]. Residue changes at the majority (17 of 29 residues) of these surface-exposed positions are located in the membrane-distal region of the globular HA1 and are likely, therefore, to influence HI (Figure 2A). Twelve of these positions are structurally equivalent to the antigenic sites recognized in human H1N1 HAs [39], and seven of these twelve, including 140 and 141 (structurally equivalent to the Ca antigenic site), 154, 155, 156 and 162 (Sa antigenic site), and 189 (Sb antigenic site) contain multiple substitutions and represent the predominant sites for antigenic variability among these viruses. Significant differences in the size and charge of amino acid side chains at positions 140, 141 and 189 at the exposed membrane-distal tip of the globular HA1 domain produce distinct conformational arrangements in all four HA structures (illustrated in Figure S2). Furthermore, the deletion of Leu129 in Egypt10 may produce a distinct antigenic surface in the 130-loop of this virus. Notably, this configuration of residues in the 130-loop is a characteristic conserved among nearly all contemporary viruses in clade 2.2.1 (termed 2.2.1 group C), as well as a group of viruses in clade 2.2. Substitutions at 12 positions located within the vestigial esterase domain and elsewhere are distant from the RBS and are, therefore, unlikely to significantly contribute to the antigenic nature of these viruses in the context of HI reactivity (Figure 2A). As observed in all other influenza viruses, the HA2 component is more highly-conserved than the HA1 of these H5N1 viruses, with comparatively little structural diversity and only three residue substitutions among all four clades, which are unlikely to influence HI reactivity.

Hence, the postulated dominant antigenic structures among these four H5N1 viruses are, approximately, spatially equivalent to established antigenic sites of H1N1 viruses in humans (Figure 2B) [39]. Previous studies to establish the primary antigenic determinants of H5 viruses among wild and domestic birds identified a number of positions in and around the H1-equivalent sites described here, by the generation of escape mutants [40], as well as algorithm-based modeling of predictive antigenic sites [41]. To assess the extent of variability at these antigenically significant positions, we compared the equivalent H1N1 antigenic sites to the consensus sequences of viruses from all 32 currently recognized H5 clades (Figure 2B). Evidence for diversifying selective pressure on this 50 residue subset of the exposed membrane-distal region of HA1 is shown by the large number of substitutions within both contemporary and historic H5N1 viruses.

### Sialic Acid Binding Properties

**Figure 3:**
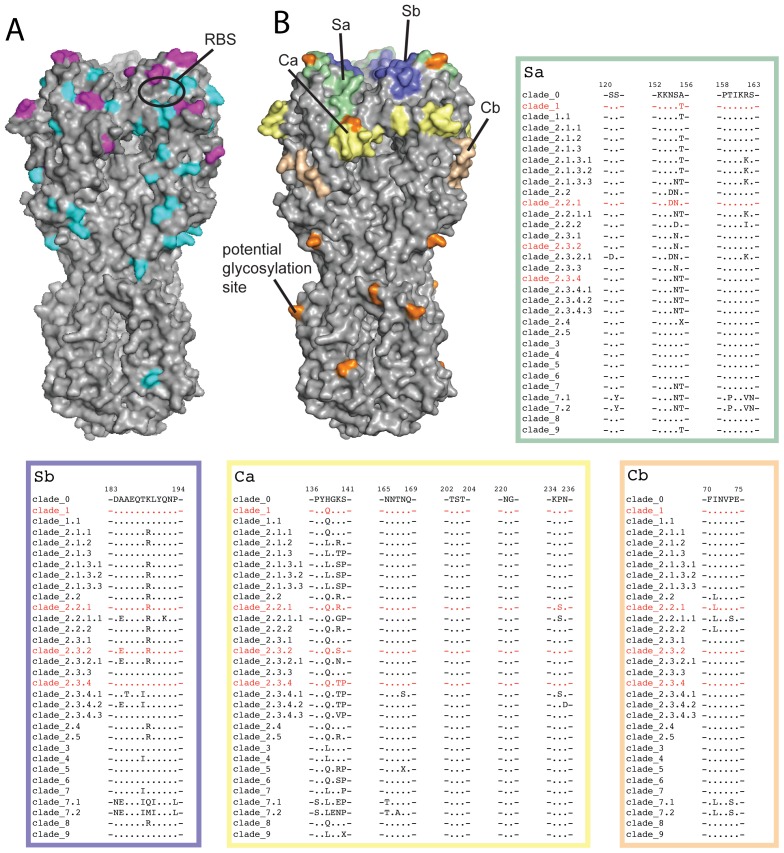
Receptor specificity of H5 recHAs. Glycan microarray analysis of recombinant Viet04 HA (A), Anhui05 (B), Egypt10 (C) and Hubei10 (D). Colored bars highlight glycans that contain α2–3 Neu5Ac (blue) and α2–6 Neu5Ac (red), α2–6/α2–3 mixed Neu5Ac (purple), N-glycolyl Neu5Ac (green), α2–8 Neu5Ac (brown), β2–6 and 9-O-acetyl Neu5Ac (yellow), and non-Neu5Ac (grey). Error bars reflect the standard error in the signal for six independent replicates on the array. The structures of each of the numbered glycans are found in Table S5.

Specificity of H5 HA for distinct linkages of sialic acid receptors at the surface of epithelial cells is widely considered a major determinant of host range and inter-species transmission [27]. Multiple studies have identified residue substitutions within and around the receptor-binding site of H5 viruses that, either alone or in combination with other mutations, were reported to increase the infectivity for host cells bearing the human-type Neu5Ac-α2-6-Gal linkage, as well as transmissibility and/or pathogenesis in mammalian models [3], [26], [28], [42]–[52]. Molecular analyses of glycan recognition by multiple HA subtypes have revealed a complicated interaction between sialylated multi-antennary oligosaccharides and influenza viruses [53]. Advances in glycan array technology allow the assessment of viral binding specificity to over 400 types of carbohydrates, including more than 50 comprising either α2–3 or α2-6-linked sialic acids [54]. Previously, this technology was used to demonstrate that the carbohydrate-binding profile of Viet04 HA could be significantly varied through mutation of residues within the receptor-binding site [36]. Anhui05, Egypt10 and Hubei10 all contain distinct residue substitutions within the region of the sialic acid binding site relative to Viet04, including substitutions that have been associated with an increase in H5 viral recognition of α2–6 linkages (Figure 1C). Ala133 (present in Hubei10) has been reported to enhance recognition of Neu5Ac-α2-6-Gal [50], and the adjacent Ala134Val substitution (also present Egypt10) was found to reduce preferential recognition of Neu5Ac-α2-3-Gal to levels equivalent to Neu5Ac-α2-6-Gal [47]. An increase in Neu5Ac-α2-6 binding has been associated with Lys189Arg (present in both Hubei10 and Egypt10) [48], and the deletion of Leu129 coupled with the substitution Ile151Thr (present in Egypt10), has been shown to increase viral binding to Neu5Ac-α2-6-Gal and infection in the airway epithelia of humans [3]. To assess the combined effect of these residue differences on receptor specificity, we subjected these three H5 recHAs to glycan microarray analysis and compared them to the recHA from Viet04. Consistent with previous findings, the glycan-binding profile of Viet04 confirmed preference for avian (α2–3) linkages, including good affinity for glycans with sulfate on the 6 position of the GlcNAc residue at the third position in the glycan chain (Figure 3A, Table S5, glycan #5–8). While Anhui05 and Hubei10 both bound to α2–3 glycans in a comparable, but weaker profile to Viet04, Egypt10 revealed a much-reduced binding profile to α2–3 glycans, with the strongest signals coming from sulfated (#5–8) and fucosylated (#30–34) α2–3 sialosides (Figure 3B, 3C & 3D, Table S5). Binding to human α2–6 receptors was minimal for all 4 recHAs tested; however, a weak binding to α2–6 sialylated *N*, *N*-diacetyllactosediamine (LacDiNAc) (glycan #56) was observed for Viet04 and Hubei10 (Figure 3A, 3D and Table S5), while additional weak binding to α2–6 biantennary glycans (#42–45) was observed for Hubei10, with some minimal interaction observed for Viet04, Egypt10 and Anhui05.

The observed binding of the panel of HAs to α2–6 LacDiNAc was further analyzed by Bio-Layer Interferometry (BLI). This label free technology was used to measure recombinant HA binding to biotinylated glycans, Neu5Ac(α2–3) Gal(β1–4) Glc-biotin (3SLN-b), Neu5Ac(α2–3)-Gal(β1–4)-GlcNAc(β1–3)-Gal(β1–4)-GlcNAcb-biotin (3SLNLN-b) and Neu5Ac(α2–6)-Gal(β1–4)-GlcNAc(β1–3)-Gal(β1–4)-GlcNAcb-biotin (6SLNLN-b) preloaded onto streptavidin coated biosensors. Results for both Viet04 and Anhui05 proteins confirmed those of the glycan microarray, in that binding to 6SLNLN-b could be detected in real time (Figure S3). The weaker binding signals observed for 6SLNLN-b in the microarrays (Figures 3A & 3D) can be explained by the higher dissociation rates (1.6×−2.5×) compared to 3SLNLN-b (Table S6). Thus, despite these HAs having a number of changes that were previously reported to increase viral attachment to human-type receptors, the combinations of these changes in Anhui05, Hubei10 and Egypt10 do not appear to increase binding to human-type (α2–6) sialic acid linkages under these experimental conditions.

## Discussion

Ongoing surveillance of HPAI H5N1 viruses has facilitated the early detection of changes in emerging strains, and significant efforts have been made to identify changes among the increasingly diverse pool of viral genotypes that may lead to increased host range and/or pathogenesis [55]. In view of the increasing diversity among circulating strains, interpretation of the antigenic changes observed in novel HA genes is important to ensure pre-pandemic vaccine candidates will offer sufficient protection in the event of a H5N1 pandemic. This study provides important insight into how changes in the receptor-binding site of circulating H5N1 viruses correlate to receptor specificity and identifies antigenically dominant structures at the surface of the H5 HA.

A primary significance of this study lies in the interpretation of how the evolution of H5N1 HPAI viruses relates to changes in antigenicity. Our data highlight the fact that the phylogenetic grouping of H5 viruses into distinct clades based on similarities at the amino acid sequence level is inadequate by itself for approximating antigenic relatedness, as residues that determine antigenicity are localized compared to the longer sequence data used in clade assignment. [56]. Moreover, structures of the antigenically distinct H5 HAs presented here enabled the identification of the dominant antigenic structures, at positions 140, 141, 154–156, 162 and 189, which contribute to the strain-specific antigenic profiles of these viruses. *N*-carbohydrates attached to Asn154 of Viet04 and Anhui05 mask the vicinal Sb-equivalent antigenic structures from immune recognition and represent an additional factor affecting antigenic variation among these viruses [40], [49]. The substitution Asn154Asp, which results in loss of this carbohydrate among clade 2.2.1 and 2.3.2.1 viruses, leaves the proximal residues exposed and potentially subject to selective immune pressure, thereby enhancing antigenic diversity among Egypt10 and Hubei10. Identification of these antigenic structures provides important information about antigenic diversity among circulating H5 HPAI viruses and can assist in interpreting the significance of amino acid substitutions in emerging strains.

Firstly, our data suggests that substitutions at the solvent-exposed positions 140, 141, 154–156, 162 and 189 of H5 HA correlate with changes in the antigenic signature of the virus, reminiscent of the spatially equivalent antigenic sites among H1 HAs circulating in the human population. Second, the H5 HA structures reported here represent a growing body of structural information for this important group of influenza A viruses, and as more H5 HA structures from antigenically diverse viruses become available, a more robust footprint of the primary antigenic sites will emerge to assist in identifying emerging strains with increased potential for antigenic diversity. Third, the identification of regions among H5 viruses that are highly prone to antigenic pressure may also improve genetic distance measures by differently weighting these antigenic regions when making clade determinations based on clustering of H5 viruses. Finally, these structural data could also aid in the development of bioinformatics software and/or databases to help surveillance efforts, monitoring how changes in avian circulating viruses correlate with changes which could lead to antigenic differences in human viruses.

Another contribution of this study is the comprehensive analysis of receptor specificities for three H5 HPAI recombinant HA proteins, both by glycan array and BLI techniques. An H5 HPAI capable of transmission between humans may be expected to possess substitutions within the RBS related to enhanced binding to the α2-6-linked receptor present within the human airway. None of the recHAs analyzed in this study exhibited preferential glycan-binding specificity for the α2–6 Neu5Ac linkage. Of note, clade 2.2.1 viruses that have recently emerged in Egypt exhibit many changes thought to enhance inter-species transmissibility and replication among mammalian hosts and are of particular concern [3]. Indeed, since 2009, approximately 50% of the total human cases of H5N1 infection worldwide have occurred in Egypt (see WHO website for regularly updated statistics). How might the apparently enhanced pathogenesis and inter-species transmissibility of these viruses be explained? Our receptor-binding analyses determined that Egypt10 recHA had no increase in α2–6 binding relative to the clade 1 Viet04 recHA (Figure 3C), although this HA did exhibit a substantial reduction in overall α2–3 binding, which may influence the pathogenesis or transmissibility of the Egypt10 virus.

Viruses belonging to the H1, H2 and H3 groups differ in species-specificity by as little as two amino acid substitutions [57]–[59], however, factors controlling host specificity remain largely ambiguous in the context of HPAI H5 viruses [26], [27]. Although enhanced recognition of α2–6 sialosides is required for effective transmission of HPAI viruses among humans, amino acid substitutions observed to enhance viral pathogenesis and replication in mammals are not restricted to those within the receptor-binding site [28], [60]. Several studies have identified changes in H5 HAs at a distance from the RBS associated with enhanced inter-species transmission, as well as replication and pathogenesis of HPAI viruses in non-avian hosts. Asp94Asn (present in Anhui05, Egypt10, Hubei10) and Met227Ile (present in Hubei10) are both located at a distance from the RBS and have been implicated in enhanced receptor specificity for α2–6 sialosides and enhanced viral infectivity of Viet04 in mammalian cells [28], [60]. Such changes may influence the association of HA monomers; residue substitutions in the region of the hydrophobic fusion peptide have been shown to influence the pH of membrane fusion and the overall stability of the viral HA, which may play a role in host determination. Likewise, the conserved change at Pro217Ser, located near the RBS at the monomer/monomer interface is also unlikely to contribute to antigenicity, but may be retained due to enhanced stability in the HA trimer. Notably, Pro is retained at this position among seasonal and ancestral H1 HAs, suggesting a species-specific preference.

*N*-glycosylation also represents an indirect means to alter the receptor function of H5 viruses and has been inversely correlated with the broadness of HA receptor specificity [61], [62]. Steric hindrance of HA/receptor interactions by *N*-glycans may be capable of changing HA receptor specificity for the distinct topology of α2–6 and α2–3 linked sialosides [22], [48]. Likewise, the removal of *N-*glycosylation in the region of the RBS has been associated with increased viral transmission, replication, and pathogenicity [26], [27], [49]. Finally, it must be recognized that our data relate to recHA binding to glycans on an array, where the avidity and kinetics of the interaction may differ from those at the host/viral interface. Moreover, it is also important to recognize the significance of other genetic components, which function in concert with the viral surface proteins, to potentially influence the impact of receptor-binding specificity in contributing towards the pathogenesis, infectivity and host range determination of HPAI viruses [46], [63].

## Supporting Information

Figure S1 **Vaccine H5 HA cleavage site comparison.** Sequence of (A) Anhui05, (B) Egypt10 and (C) Hubei10 H5 HAs were compared to their wild type counterparts. The polybasic cleavage site in the wild type virus sequence is boxed. Residues highlighted in cyan could not be built in the three models reported here, due to poor density in this region.
(DOCX)

Figure S2 **Structural bases for antigenic variation among vaccine candidate viruses.** Topological and electrostatic changes at positions surrounding the receptor-binding site are indicated on a molecular surface representation of (A) Viet04, (B) Anhui05, (C) Egypt10 and (D) Hubei10. The absence of Leu129 in Egypt10 (c) results in a more flattened, open conformation in the 130 loop relative to the other four viruses. The N-carbohydrate attached to Asn154 of Anhui05 (a) and Viet04 (b) has been removed for clarity.
(TIF)

Figure S3 **Kinetic** **binding analysis of (A) Viet04, (B) Anhui05, (C) Egypt10 and (D) Hubei10 recombinant HAs.** The binding kinetics to specific biotinylated glycans (3SLN-b, 3SLNLN-b, and 6SLNLN-b), immobilized onto biosensors, were analyzed by BLI (A, B, C and D).
(TIF)

Table S1 **Results of multiple HI Assays to determine cross-reactivity of Viet04, Anhui05, Egypt10 and Hubei10 against strain-specific ferret anti-sera.**
(DOCX)

Table S2 **Data collection and refinement statistics for the Anhui05, Egypt10 and Hubei10 crystal structures.**
(DOCX)

Table S3 **RMSD (Å) comparison for the HA1 and HA2 domains of each HA to previously reported clade 1, Viet04.**
(DOCX)

Table S4 **Residue differences among Anhui05, Egypt10 and Hubei10 compared to Viet04.** (A) Changes among surface residues, exposed to immune surveillance. (B) Substituted residues that do not have solvent accessible side chains. Positions within antigenic sites are asindicated.
(DOCX)

Table S5 **Glycan microarray differences between Viet04, Anhui05, Egypt10 and Hubei10.** The color coding in the left hand column reflects the same coloring scheme used in Figure 3. Significant binding of samples to glycans were qualitatively estimated based on relative strength of the signal for the data shown in the figure; Fluorescence Intensity >20000 (+++), 10000–19999 (++), 5000–9999 (+), <2500 (nb). Different categories of glycans on the array are color-coded in column 1 as follows: No color, sialic acid; blue, α2–3 sialosides; red, α2–6 sialosides, violet, mixed α2–3/α2–6 biantennaries; green, N-glycolylneuraminic acid-containing glycans; brown, α2–8 linked sialosides; pink, β2–6 linked and 9-O-acetylated sialic acids; grey, asialo glycans.
(DOCX)

Table S6 **Kinetics results for glycan binding to Viet04, Anhui05, Egypt10 and Hubei10 recombinant HAs.**
(DOCX)
