# Supplementary material for: Structural and Antigenic Variation among Diverse Clade 2 H5N1 Viruses
Source: PLoS One. 2013 Sep 27;8(9):e75209. doi: 10.1371/journal.pone.0075209 (PMC3785507; doi:10.1371/journal.pone.0075209)
Supplement: Figure S1 — Vaccine H5 HA cleavage site comparison. Sequence of (A) Anhui05, (B) Egypt10 and (C) Hubei10 H5 HAs were compared to their wild type counterparts. The polybasic cleavage site in the wild type virus sequence is boxed. Residues highlighted in cyan could not be built in the three models reported here, due to poor density in this region. (DOCX) [file pone.0075209.s001.docx]

**Figure S1**

A)

Anhui05 WT HA1..GLRNSPLRERRRKRGLFGAIAGFIEGGWQ...HA2

Anhui05 RG HA1..GLRNSPLRER----GLFGAIAGFIEGGWQ...HA2

B)

Egypt10 WT HA1..GLRNSPQGERRRKKRGLFGAIAGFIEGGWQ...HA2

Egypt10 RG HA1..GLRNSPQGETR----GLFGAIAGFIEGGWQ...HA2

C)

Hubei10 WT HA1..GLRNSPQRERRRKRGLFGAIAGFIEGGWQ...HA2

Hubei10 RG HA1..GLRNSPQRETR---GLFGAIAGFIEGGWQ...HA2
