# Supplementary figures and images for: Structural and Antigenic Variation among Diverse Clade 2 H5N1 Viruses
Source: PLoS One. 2013 Sep 27;8(9):e75209. doi: 10.1371/journal.pone.0075209 (PMC3785507; doi:10.1371/journal.pone.0075209)

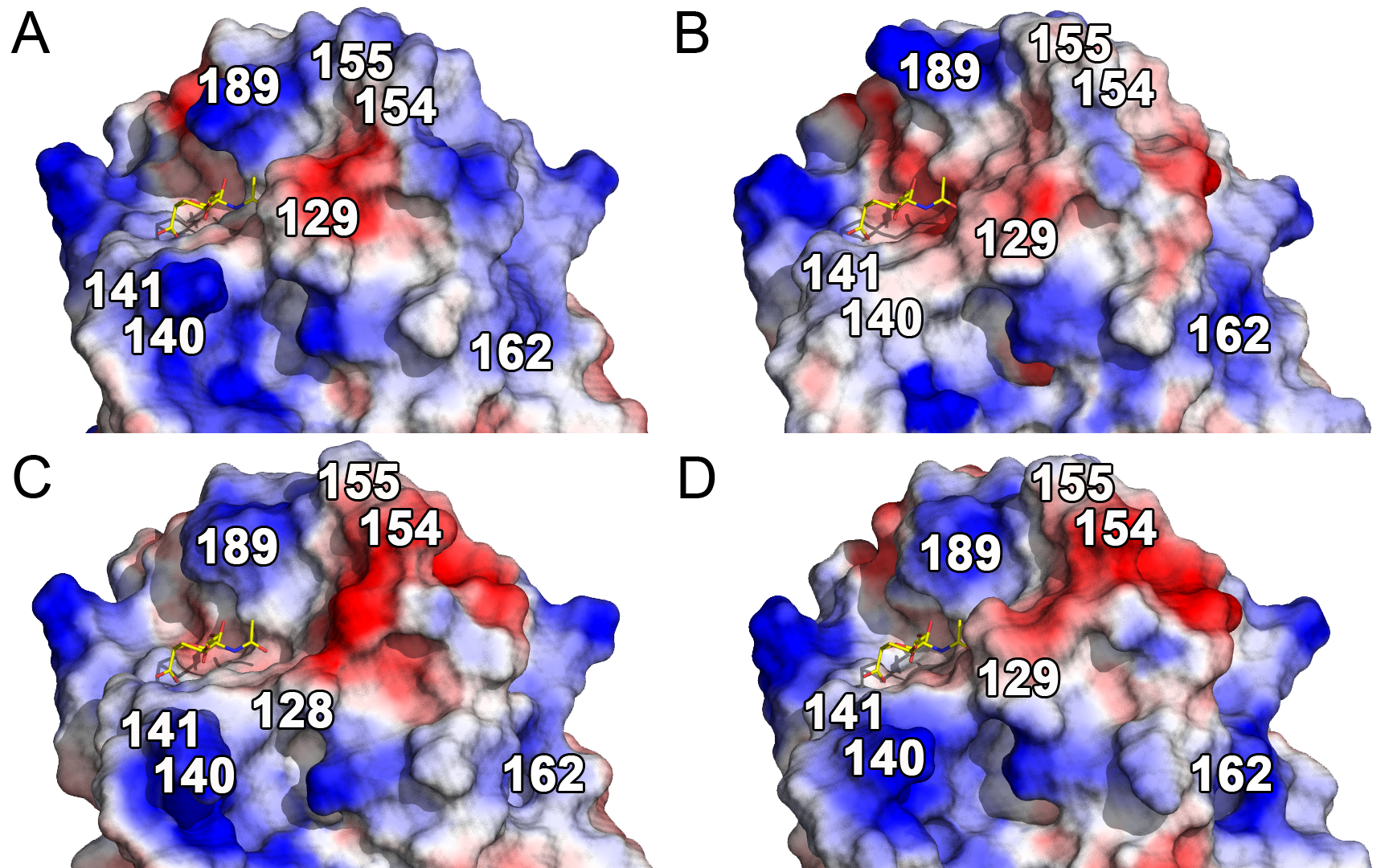

Supplement: Figure S2 — Structural bases for antigenic variation among vaccine candidate viruses. Topological and electrostatic changes at positions surrounding the receptor-binding site are indicated on a molecular surface representation of (A) Viet04, (B) Anhui05, (C) Egypt10 and (D) Hubei10. The absence of Leu129 in Egypt10 (c) results in a more flattened, open conformation in the 130 loop relative to the other four viruses. The N-carbohydrate attached to Asn154 of Anhui05 (a) and Viet04 (b) has been removed for clarity. (TIF) [file pone.0075209.s002.tif]

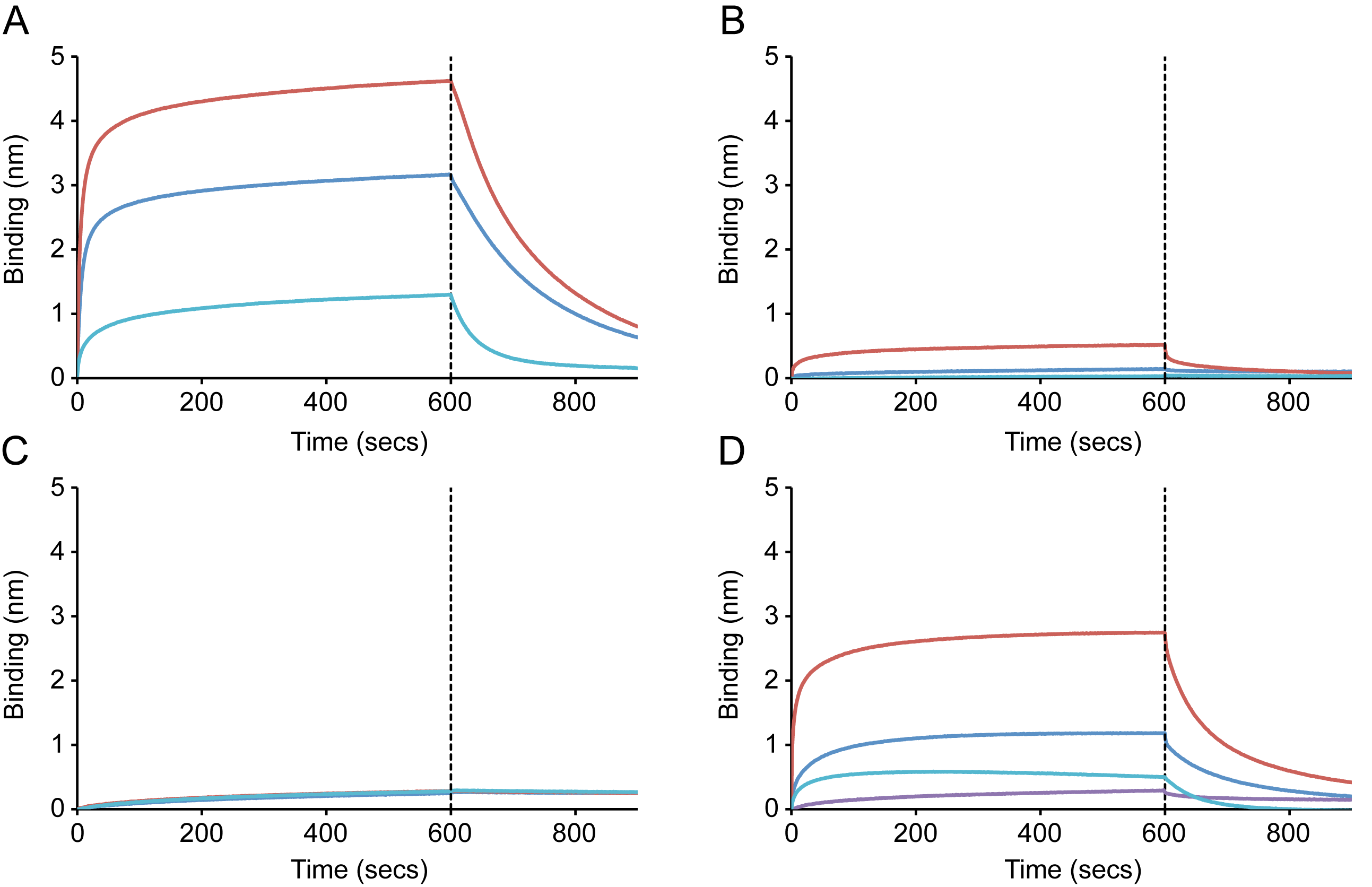

Supplement: Figure S3 — Kinetic binding analysis of (A) Viet04, (B) Anhui05, (C) Egypt10 and (D) Hubei10 recombinant HAs. The binding kinetics to specific biotinylated glycans (3SLN-b, 3SLNLN-b, and 6SLNLN-b), immobilized onto biosensors, were analyzed by BLI (A, B, C and D). (TIF) [file pone.0075209.s003.tif]
