# Supplementary material for: Structural and Antigenic Variation among Diverse Clade 2 H5N1 Viruses
Source: PLoS One. 2013 Sep 27;8(9):e75209. doi: 10.1371/journal.pone.0075209 (PMC3785507; doi:10.1371/journal.pone.0075209)
Supplement: Table S1 — Results of multiple HI Assays to determine cross-reactivity of Viet04, Anhui05, Egypt10 and Hubei10 against strain-specific ferret anti-sera. (DOCX) [file pone.0075209.s004.docx]

**Table S1.** HI reactivity among different clades of H5N1 vaccine candidate viruses; .

| Antigen | Antisera | | | |
| --- | --- | --- | --- | --- |
|  | Viet04 | Anhui05 | Egypt10 | Hubei10 |
| Viet04 | 285 ^a,b^ | 57 | 13 | 57 |
| Anhui05 | 90 | 718 | 18 | 16 |
| **Egypt10** | **57** | **285** | **3620** | **226** |
| **Hubei10 ^c^** | **20** | **22** | **101** | **285** |

**^a^ Hemagglutination inhibition (HI) titers were determined using turkey red blood cells.**

**^b^ Titers for homologous antigen/antisera are shown with values underlined. Titers are presented as the geometric mean titers (GMT) calculated from six independent HI tests**

**^c^ Reassortant virus was used in these assays.**
