# Supplementary material for: Structural and Antigenic Variation among Diverse Clade 2 H5N1 Viruses
Source: PLoS One. 2013 Sep 27;8(9):e75209. doi: 10.1371/journal.pone.0075209 (PMC3785507; doi:10.1371/journal.pone.0075209)
Supplement: Table S2 — Data collection and refinement statistics for the Anhui05, Egypt10 and Hubei10 crystal structures. (DOCX) [file pone.0075209.s005.docx]

**Table S2.** Data collection and refinement statistics for the Anhui05, Egypt10 and Hubei10 crystal structures**.**

| **Data collection** | **Anhui05** | **Egypt10** | **Hubei10** |
| --- | --- | --- | --- |
| Space group | P3 | H3 | C2 |
| Cell dimensions | 114.37 Å, 114.37 Å, 134.89 Å  90°, 90°, 120° | 98.17 Å, 98.17 Å, 655.66 Å, 90°, 90°, 120° | 174.121, 101.594, 124.94  90, 121.66, 90 |
| Resolution (Å) | 50-2.7 (2.80-2.70)^a^ | 50 - 2.5 | 50 - 2.6 (2.64 - 2.60) |
| R_sym_ (%) | 11.8 (55.9) | 7.6 (42.1) | 8.9 (64.8) |
| I/σ | 11.2 (1.7) | 10.1 (1.6) | 14.75 (1.8) |
| Completeness (%) | 100 (100) | 98.6 (100) | 99.6 (100) |
| Redundancy | 2.9 (2.9) | 2.1 (2.1) | 3.7 (3.8) |
| **Refinement** |  |  |  |
| Resolution (Å) | 46.5-2.7 (2.77-2.70) | 218.7-2.5 (2.56-2.50) | 106.3-2.6 (2.66-2.6) |
| No. of reflections (total) | 51513 | 77003 | 53820 (3741) |
| No. of reflections (test) | 2759 | 3980 | 2876 |
| R_work_/ R_free_ | 24.1/28.6 | 22.1/25.3 | 23.4/26.6 |
| No. of atoms | 11853 | 15701 | 11987 |
| r.m.s.d.- bond length (Å) | 0.008 | 0.010 | 0.006 |
| r.m.s.d.- bond angle (°) | 1.287 | 1.660 | 1.073 |
| **MolProbity scores^b^** |  |  |  |
| Favored (%) | 91.7 | 94.9 | 95.2 |
| Allowed (%) | 99.2 | 99.5 | 99.6 |
| Outliers (%) _(No. of residues)_ | 0.8 _(12/1451)_ | 0.5 _(10/1872)_ | 0.4 _(6/1450)_ |
| PDB code | AAAA | BBBB | CCCC |

^a^ Numbers in parentheses refer to the highest resolution shell.

^b^ Reference: Davis et al., 2007
