# Supplementary material for: Structural and Antigenic Variation among Diverse Clade 2 H5N1 Viruses
Source: PLoS One. 2013 Sep 27;8(9):e75209. doi: 10.1371/journal.pone.0075209 (PMC3785507; doi:10.1371/journal.pone.0075209)
Supplement: Table S3 — RMSD (Å) comparison for the HA1 and HA2 domains of each HA to previously reported clade 1, Viet04. (DOCX) [file pone.0075209.s006.docx]

**Table S3**

|  | Superpose onto Viet04: | Monomer | HA1 domain | HA2 domain | |
| --- | --- | --- | --- | --- | --- |
| Clade | Analyze differences between^a^ | Monomers | HA1 domains | HA1 domains | HA2 domains |
| 2.3.4 | Anhui05 | 1.04 | 1.24 | 2.69 | 0.92 |
| 2.2.1 | Egypt10 | 0.85 | 0.67 | 2.08 | 1.49 |
| 2.3.2 | Hubei10 | 0.74 | 0.57 | 2.22 | 1.38 |

^a^ For analyzing differences in the overall structure, RMSD values (Å) were calculated between monomers or sub-domains of different HAs, after the Cα atoms of the indicated domain were superposed by sequence and structural alignment onto the equivalent domain of Viet04.
