# Supplementary material for: Structural and Antigenic Variation among Diverse Clade 2 H5N1 Viruses
Source: PLoS One. 2013 Sep 27;8(9):e75209. doi: 10.1371/journal.pone.0075209 (PMC3785507; doi:10.1371/journal.pone.0075209)
Supplement: Table S4 — Residue differences among Anhui05, Egypt10 and Hubei10 compared to Viet04. (A) Changes among surface residues, exposed to immune surveillance. (B) Substituted residues that do not have solvent accessible side chains. Positions within antigenic sites are asindicated. (DOCX) [file pone.0075209.s007.docx]

**Table S4**

A

| **Virus** | **Clade** | **Residue at amino acid position** | | | | | | | | | | | | | | | | | | | | | | | | | | | | |
| --- | --- | --- | --- | --- | --- | --- | --- | --- | --- | --- | --- | --- | --- | --- | --- | --- | --- | --- | --- | --- | --- | --- | --- | --- | --- | --- | --- | --- | --- | --- |
|  |  | **2** | **36** | **43** | **45** | **53** | **83** | **86** | **120** | **124** | **129** | **133** | **134** | **140** | **141** | **151** | **154** | **155** | **156** | **162** | **163** | **184** | **189** | **219** | **235** | **240** | **269** | **277** | **282** | **310** |
| Vietnam/1203/04 | 1 | **Q** | **K** | **D** | **D** | **R** | **A** | **V** | **S** | **S** | **L** | **S** | **A** | **K** | **S** | **I** | **N** | **S** | **T** | **R** | **S** | **A** | **K** | **V** | **P** | **N** | **L** | **K** | **M** | **R** |
| Anhui/5/05 | 2.3.4 |  | **T** |  |  |  |  | **A** |  | **D** | **S** |  |  | **T** | **P** |  |  | **N** |  |  |  |  |  |  |  |  | **V** |  | **I** | **K** |
| Egypt/N03072/10 | 2.2.1 |  | **T** | **N** |  |  | **I** | **A** |  | **D** | **/** |  | **V** | **R** |  | **T** | **D** | **N** | **A** |  |  |  | **R** |  | **S** |  |  |  | **I** |  |
| Hubei/1/10 | 2.3.2 | **H** | **T** |  | **N** | **K** |  | **A** | **N** | **D** |  | **A** |  |  |  |  | **D** | **N** | **A** | **K** | **G** | **E** | **R** | **I** |  | **H** | **V** | **R** | **I** | **K** |
| Antigenic site |  |  |  |  |  |  |  |  | **Sa** |  |  |  |  | **Ca** | **Ca** |  | **Sa** | **Sa** | **Sa** | **Sa** | **Sa** | **Sb** | **Sb** |  | **Ca** |  |  |  |  |  |

B

| **Virus** | **Clade** | **Residue at amino acid position** | | | | | | | | | | | |
| --- | --- | --- | --- | --- | --- | --- | --- | --- | --- | --- | --- | --- | --- |
|  |  | **71** | **94** | **174** | **181** | **200** | **212** | **217** | **226** | **227** | **252** | **263** | **265** |
| Vietnam/1203/2004 | 1 | I | D | V | P | V | R | S | M | E | Y | T | M |
| Anhui/5/2005 | 2.3.4 |  | N | I | S |  | K | S |  | D |  | A | V |
| Egypt/N03072/2010 | 2.2.1 | L | N |  |  |  | K | S |  |  | N |  |  |
| Hubei/1/2010 | 2.3.2 |  | N |  |  | I | K | S | I | D |  |  |  |
| Antigenic site |  | Cb |  |  |  |  |  |  |  |  |  |  |  |
