# Supplementary material for: Structural and Antigenic Variation among Diverse Clade 2 H5N1 Viruses
Source: PLoS One. 2013 Sep 27;8(9):e75209. doi: 10.1371/journal.pone.0075209 (PMC3785507; doi:10.1371/journal.pone.0075209)
Supplement: Table S5 — Glycan microarray differences between Viet04, Anhui05, Egypt10 and Hubei10. The color coding in the left hand column reflects the same coloring scheme used in Figure 3. Significant binding of samples to glycans were qualitatively estimated based on relative strength of the signal for the data shown in the figure; Fluorescence Intensity >20000 (+++), 10000–19999 (++), 5000–9999 (+), <2500 (nb). Different categories of glycans on the array are color-coded in column 1 as follows: No color, sialic acid; blue, α2–3 sialosides; red, α2–6 sialosides, violet, mixed α2–3/α2–6 biantennaries; green, N-glycolylneuraminic acid-containing glycans; brown, α2–8 linked sialosides; pink, β2–6 linked and 9-O-acetylated sialic acids; grey, asialo glycans. (DOCX) [file pone.0075209.s008.docx]

**Table S5**

| **#** | **Structure** | **Viet04** | **Anhui05** | **Egypt10** | **Hubei10** |
| --- | --- | --- | --- | --- | --- |
| 1 | Neu5Acα | nb | nb | nb | nb |
| 2 | Neu5Acα | nb | nb | nb | nb |
| 3 | Neu5Acβ | nb | nb | nb | nb |
| 4 | Neu5Acα2-3(6-O-Su)Galβ1-4GlcNAcβ | +++ | +++ | nb | +++ |
| 5 | Neu5Acα2-3Galβ1-3[6OSO3]GalNAcα | +++ | +++ | ++ | +++ |
| 6 | Neu5Acα2-3Galβ1-4[6OSO3]GlcNAcβ | +++ | ++ | ++ | +++ |
| 7 | Neu5Acα2-3Galβ1-4(Fucα1-3)[6OSO3]GlcNAcβ-propyl-NH2 | +++ | ++ | ++ | +++ |
| 8 | Neu5Acα2-3Galβ1-3[6OSO3]GlcNAc-propyl-NH2 | +++ | ++ | ++ | +++ |
| 9 | Neu5Acα2-3Galβ1-3(Neu5Acα2-3Galβ1-4)GlcNAcβ | +++ | ++ | ++ | +++ |
| 10 | Neu5Acα2-3Galβ1-3(Neu5Acα2-3Galβ1-4GlcNAcβ1-6)GalNAcα | +++ | ++ | ++ | +++ |
| 11 | Neu5Acα2-3Galβ1-4GlcNAcβ1-2Manα1-3(Neu5Acα2-3Galβ1-4GlcNAcβ1-2Manα1-6)Manβ1-4GlcNAcβ1-4GlcNAcβ | +++ | ++ | + | ++ |
| 12 | NeuAcα(2-3)-Galβ(1-4)-GlcNAcβ(1-3)-Galβ(1-4)-GlcNAcβ(1-2)-Manα(1-3)-[NeuAcα(2-3)-Galβ(1-4)-GlcNAcβ(1-3)-Galβ(1-4)-GlcNAcβ(1-2)-Manα(1-6)]-Manβ(1-4)-GlcNAcβ(1-4)-GlcNAcβ | +++ | ++ | + | ++ |
| 13 | Neu5Acα2-3Galβ | +++ | +++ | nb | +++ |
| 14 | Neu5Acα2-3Galβ1-3GalNAcα | +++ | +++ | + | +++ |
| 15 | Neu5Acα2-3Galβ1-3GlcNAcβ | +++ | +++ | nb | +++ |
| 16 | Neu5Acα2-3Galβ1-3GlcNAcβ | +++ | +++ | nb | +++ |
| 17 | Neu5Acα2-3Galβ1-4Glcβ | +++ | + | nb | ++ |
| 18 | Neu5Acα2-3Galβ1-4Glcβ | +++ | +++ | nb | +++ |
| 19 | Neu5Acα2-3Galβ1-4GlcNAcβ | +++ | ++ | + | +++ |
| 20 | Neu5Acα2-3Galβ1-4GlcNAcβ | +++ | ++ | + | +++ |
| 21 | Neu5Acα2-3GalNAcβ1-4GlcNAcβ | +++ | ++ | nb | ++ |
| 22 | Neu5Acα2-3Galβ1-4GlcNAcβ1-3Galβ1-4GlcNAcβ | +++ | +++ | + | ++ |
| 23 | NeuAcα2,3Galβ1-3GlcNAcβ1-3Galβ1-4GlcNAcβ | +++ | ++ | + | +++ |
| 24 | Neu5Acα2-3Galβ1-4GlcNAcβ1-3Galβ1-4GlcNAcβ1-3Galβ1-4GlcNAcβ | +++ | ++ | nb | ++ |
| 25 | Neu5Acα2-3Galβ1-4GlcNAcβ1-3Galβ1-3GlcNAcβ | +++ | ++ | nb | +++ |
| 26 | Neu5Acα2-3Galβ1-3GalNAcα | +++ | ++ | nb | +++ |
| 27 | Galβ1-3(Neu5Acα2-3Galβ1-4(Fucα1-3)GlcNAcβ1-6)GalNAcα | nb | nb | nb | nb |
| 28 | Neu5Acα2-3Galβ1-3(Fucα1-4)GlcNAcβ | +++ | ++ | nb | +++ |
| 29 | Neu5Acα2-3Galβ1-4(Fucα1-3)GlcNAcβ | nb | nb | nb | nb |
| 30 | Neu5Acα2-3Galβ1-4(Fucα1-3)GlcNAcβ | +++ | ++ | +++ | +++ |
| 31 | Neu5Acα2-3Galβ1-4(Fucα1-3)GlcNAcβ1-3Galβ | +++ | +++ | + | +++ |
| 32 | NeuAcα2-3Galβ1-3[Fucα1-4]GlcNAcβ1-3Galβ1-4[Fucα1-3]GlcNAcβ | +++ | +++ | nb | +++ |
| 33 | NeuAcα2-3Galβ1-3[Fucα1-3]GlcNAcβ1-3Galβ1-4[Fucα1-3]GlcNAcβ | +++ | ++ | ++ | ++ |
| 34 | Neu5Acα2-3Galβ1-4(Fucα1-3)GlcNAcβ1-3Galβ1-4(Fucα1-3)GlcNAcβ1-3Galβ1-4(Fucα1-3)GlcNAcβ | +++ | ++ | ++ | +++ |
| 35 | Neu5Acα2-3(GalNAcβ1-4)Galβ1-4GlcNAcβ | nb | nb | nb | nb |
| 36 | Neu5Acα2-3(GalNAcβ1-4)Galβ1-4GlcNAcβ | nb | nb | nb | nb |
| 37 | Neu5Acα2-3(GalNAcβ1-4)Galβ1-4Glcβ | nb | nb | nb | nb |
| 38 | Galβ1-3GalNAcβ1-4(Neu5Acα2-3)Galβ1-4Glcβ | nb | nb | nb | nb |
| 39 | Fucα1-2Galβ1-3GalNAcβ1-4(Neu5Acα2-3)Galβ1-4Glcβ | nb | nb | nb | nb |
| 40 | Fucα1-2Galβ1-3GalNAcβ1-4(Neu5Acα2-3)Galβ1-4Glcβ | nb | nb | nb | nb |
| 41 | Neu5Acα2-6Galβ1-4[6OSO3]GlcNAcβ | nb | nb | nb | nb |
| 42 | Neu5Acα2-6Galβ1-4GlcNAcβ1-2Manα1-3(Galβ1-4GlcNAcβ1-2Manα1-6)Manβ1-4GlcNAcβ1-4GlcNAcβ | nb | nb | nb | nb |
| 43 | Neu5Acα2-6Galβ1-4GlcNAcβ1-2Manα1-3(Neu5Acα2-6Galβ1-4GlcNAcβ1-2Manα1-6)Manβ1-4GlcNAcβ1-4GlcNAcβ | nb | nb | nb | nb |
| 44 | NeuAcα2-6Galβ1-4GlcNAcβ1-3Galβ1-4GlcNAcβ1-2Manα1-3[NeuAcα2-6Galβ1-4GlcNAcβ1-3Galβ1-4GlcNAcβ1-2Manα1-6]Manβ1-4GlcNAcβ1-4GlcNAcβ | nb | nb | nb | + |
| 45 | NeuAcα2-6Galβ1-4GlcNAcβ1-3Galβ1-4GlcNAcβ1-3Galβ1-4GlcNAcβ1-2Manα1-3[NeuAcα2-6Galβ1-4GlcNAcβ1-3Galβ1-4GlcNAcβ1-3Galβ1-4GlcNAcβ1-2Manα1-6]-Manβ1-4GlcNAcβ1-4GlcNAcβ | + | nb | nb | ++ |
| 46 | NeuAcα2-6Galβ1-4GlcNAcβ1-3Galβ1-4GlcNAcβ1-3[NeuAcα2-6Galβ1-4GlcNAcβ1-3Galβ1-4GlcNAcβ1-6]GalNAca | nb | nb | nb | nb |
| 47 | NeuAcα2-6Galβ1-4GlcNAcβ1-3[NeuAcα2-6Galβ1-4GlcNAcβ1-6]GalNAca | nb | nb | nb | nb |
| 48 | Neu5Acα2-6GalNAcα | nb | nb | nb | nb |
| 49 | Neu5Acα2-6Galβ | nb | nb | nb | nb |
| 50 | Neu5Acα2-6Galβ1-4Glcβ | nb | nb | nb | nb |
| 51 | Neu5Acα2-6Galβ1-4Glcβ | nb | nb | nb | nb |
| 52 | Neu5Acα2-6Galβ1-4GlcNAcβ | nb | nb | nb | nb |
| 53 | Neu5Acα2-6Galβ1-4GlcNAcβ | nb | nb | nb | nb |
| 54 | Neu5Acα2-6GalNAcβ1-4GlcNAcβ | nb | nb | nb | nb |
| 55 | NeuAcα2-6Galβ1-4GlcNAcβ1-3GalNAcα | nb | nb | nb | nb |
| 56 | Neu5Acα2-6Galβ1-4GlcNAcβ1-3Galβ1-4GlcNAcβ | ++ | nb | nb | + |
| 57 | NeuAcα2-6Galβ1-4GlcNAcβ1-3Galβ1-4GlcNAcβ1-3GalNAcα | nb | nb | nb | nb |
| 58 | NeuAcα2,6Galβ1-4GlcNAcβ1-3Galβ1-4GlcNAcβ1-3Galβ1-4GlcNAcβ | nb | nb | nb | nb |
| 59 | Neu5Acα2-6Galβ1-4GlcNAcβ1-3Galβ1-4(Fucα1-3)GlcNAcβ1-3Galβ1-4(Fucα1-3)GlcNAcβ | nb | nb | nb | nb |
| 60 | Galβ1-3(Neu5Acα2-6)GlcNAcβ1-4Galβ1-4Glcβ-Sp10 | nb | nb | nb | nb |
| 61 | NeuAcα2-6[Galβ1-3]GalNAca | nb | nb | nb | nb |
| 62 | NeuAcα2-6Galβ1-4GlcNAcβ1-6[Galβ1-3]GalNAca | nb | nb | nb | nb |
| 63 | NeuAcα2-6Galβ1-4GlcNAcβ1-3Galβ1-4GlcNAcβ1-6[Galβ1-3]GalNAca | nb | nb | nb | nb |
| 64 | Neu5Acα2-3Galβ1-4GlcNAcβ1-2Manα1-3(Neu5Acα2-6Galβ1-4GlcNAcβ1-2Manα1-6)Manβ1-4GlcNAcβ1-4GlcNAcβ | nb | nb | nb | nb |
| 65 | Neu5Acα2-6Galβ1-4GlcNAcβ1-2Manα1-3(Neu5Acα2-3Galβ1-4GlcNAcβ1-2Manα1-6)Manβ1-4GlcNAcβ1-4GlcNAcβ | +++ | ++ | nb | ++ |
| 66 | Neu5Acα2-3Galβ1-3(Neu5Acα2-6)GalNAcα | +++ | ++ | ++ | +++ |
| 67 | Neu5Acα2-3(Neu5Acα2-6)GalNAcα | +++ | nb | nb | ++ |
| 68 | Neu5Gcα | nb | nb | nb | nb |
| 69 | Neu5Gcα2-3Galβ1-3(Fucα1-4)GlcNAcβ | nb | nb | nb | nb |
| 70 | Neu5Gcα2-3Galβ1-3GlcNAcβ | nb | nb | nb | nb |
| 71 | Neu5Gcα2-3Galβ1-4(Fucα1-3)GlcNAcβ | +++ | ++ | nb | ++ |
| 72 | Neu5Gcα2-3Galβ1-4GlcNAcβ | nb | nb | nb | nb |
| 73 | Neu5Gcα2-6GalNAcα | nb | nb | nb | nb |
| 74 | Neu5Gcα2-6Galβ1-4GlcNAcβ | nb | nb | nb | nb |
| 75 | Neu5Acα2-8Neu5Acα | nb | nb | nb | nb |
| 76 | Neu5Acα2-8Neu5Acα2-8Neu5Acα | nb | nb | nb | nb |
| 77 | Neu5Acα2-8Neu5Acα2-3(GalNAcβ1-4)Galβ1-4Glcβ | nb | nb | nb | nb |
| 78 | Neu5Acα2-8Neu5Acα2-3Galβ1-4Glcβ | nb | nb | nb | nb |
| 79 | Neu5Acα2-8Neu5Acα2-8Neu5Acα2-3(GalNAcβ1-4)Galβ1-4Glcβ | nb | nb | nb | nb |
| 80 | Neu5Acα2-8Neu5Acα2-8Neu5Acα2-3Galβ1-4Glcβ | nb | nb | nb | nb |
| 81 | Neu5Acα2-8Neu5Acβ-Sp17 | nb | nb | nb | nb |
| 82 | Neu5Acα2-8Neu5Acα2-8Neu5Acβ | nb | nb | nb | nb |
| 83 | Neu5Acβ2-6GalNAcα | nb | nb | nb | nb |
| 84 | Neu5Acβ2-6Galβ1-4GlcNAcβ | nb | nb | nb | nb |
| 85 | Neu5Gcβ2-6Galβ1-4GlcNAc | nb | nb | nb | nb |
| 86 | Galβ1-3(Neu5Acβ2-6)GalNAcα | nb | nb | nb | nb |
| 87 | [9NAc]Neu5Acα | nb | nb | nb | nb |
| 88 | [9NAc]Neu5Acα2-6Galβ1-4GlcNAcβ | nb | nb | nb | nb |
| 89 | Galβ1-4GlcNAcβ1-3Galβ1-4GlcNAcβ1-3Galβ1-4GlcNAcβ | nb | nb | nb | nb |
| 90 | Galβ1-3GlcNAcβ1-3Galβ1-3GlcNAcβ | nb | nb | nb | nb |
| 91 | Galβ1-4GlcNAcβ1-2Manα1-3[Galβ1-4GlcNAcβ1-2Manα1-6]Manβ1-4GlcNAcβ1-4GlcNAcβ | nb | nb | nb | nb |
| 92 | GalNAcα1-3(Fucα1-2)Galβ1-3GlcNAcβ | nb | nb | nb | nb |
| 93 | GalNAcα1-3(Fucα1-2)Galβ1-4GlcNAcβ | nb | nb | nb | nb |
| 94 | Galα1-3(Fucα1-2)Galβ1-3GlcNAcβ | nb | nb | nb | nb |
| 95 | Galα1-3(Fucα1-2)Galβ1-4(Fucα1-3)GlcNAcβ | nb | nb | nb | nb |
| 96 | Galβ1-3GalNAcα | nb | nb | nb | nb |
