# Supplementary material for: Structural and Antigenic Variation among Diverse Clade 2 H5N1 Viruses
Source: PLoS One. 2013 Sep 27;8(9):e75209. doi: 10.1371/journal.pone.0075209 (PMC3785507; doi:10.1371/journal.pone.0075209)
Supplement: Table S6 — Kinetics results for glycan binding to Viet04, Anhui05, Egypt10 and Hubei10 recombinant HAs. (DOCX) [file pone.0075209.s009.docx]

**Table S6**

| **Protein** | **Glycan** | **Apparent *K*_D_ (µM)** | ***k_a_* (x10^4^ 1/Ms)** | ***k_d_ (x10^-3^ 1/s)*** | ***k_d_ error (x10^-3^ 1/s)*** | ***k_obs_ (1/s)*** | ***k_obs_ error (1/s)*** |
| --- | --- | --- | --- | --- | --- | --- | --- |
| Viet04 | 3SLN-b | 0.301 | 1.87 | 5.62 | 0.008 | 0.093 | 0.0012 |
|  | 3SLNLN-b | 0.262 | 2.37 | 6.22 | 0.013 | 0.117 | 0.00018 |
|  | 6SLNLN-b | 1.22 | 0.82 | 10 | 0.105 | 0.048 | 0.0007 |
| Anhui05 | 3SLN-b | NB# | --- | --- | --- | --- | --- |
|  | 3SLNLN-b | 0.491 | 1.23 | 6.04 | 0.07 | 0.063 | 0.0016 |
|  | 6SLNLN-b | NB# | --- | --- | --- | --- | --- |
| Egypt10 | 3SLN-b | NB# | --- | --- | --- | --- | --- |
|  | 3SLNLN-b | NB# | --- | --- | --- | --- | --- |
|  | 6SLNLN-b | NB# | --- | --- | --- | --- | --- |
| Hubei10 | 3SLN-b | 0.983 | 0.69 | 6.81 | 0.032 | 0.042 | 0.0006 |
|  | 3SLNLN-b | 0.349 | 2.27 | 7.93 | 0.048 | 0.124 | 0.0027 |
|  | 6SLNLN-b | 2.94 | 0.68 | 19.9 | 0.026 | 0.055 | 0.0007 |

^#^ No binding
